# LZ-207, a Newly Synthesized Flavonoid, Induces Apoptosis and Suppresses Inflammation-Related Colon Cancer by Inhibiting the NF-κB Signaling Pathway

**DOI:** 10.1371/journal.pone.0127282

**Published:** 2015-05-29

**Authors:** Jie Sun, Fanni Li, Yue Zhao, Li Zhao, Chen Qiao, Zhiyu Li, Qinglong Guo, Na Lu

**Affiliations:** 1 State Key Laboratory of Natural Medicines, Jiangsu Key Laboratory of Carcinogenesis and Intervention, China Pharmaceutical University, 24 Tongjiaxiang, Nanjing 210009, People’s Republic of China; 2 School of Pharmacy, China Pharmaceutical University, Nanjing 210009, People’s Republic of China; UMR INSERM U866, FRANCE

## Abstract

Flavonoids and flavonoid derivatives, which have significant biological and pharmacological activities, including antitumor and anti-inflammatory activities, have been widely used in human healthcare. To design a more effective flavonoid antitumor agent, we altered the flavonoid backbone with substitutions of piperazine and methoxy groups to synthesize a novel flavonoid derivative, LZ-207. The anticancer effect of LZ-207 against HCT116 colon cancer cells and the underlying mechanism of this effect were explored in this study. Specifically, LZ-207 exhibited inhibitory effects on growth and viability in several human colon cancer cell lines and induced apoptosis in HCT116 cells both in vitro and in vivo. LZ-207 treatment also suppressed the nuclear translocation of NF-κB and the phosphorylation of IκB and IKKα/β in a dose-dependent manner in both HCT116 cells and human acute monocytic leukemia THP-1 cells. Moreover, LZ-207 also reduced the secretion of the pro-inflammatory cytokine interleukin-6 (IL-6) in LPS-induced THP-1 cells, and this effect was confirmed at the transcriptional level. Furthermore, LZ-207 significantly inhibited HCT116 cell proliferation that was elicited by LPS-induced THP-1 cells in a co-culture system. These findings elucidated some potential molecular mechanisms for preventing inflammation-driven colon cancer using the newly synthesized flavonoid LZ-207 and suggested the possibility of further developing novel therapeutic agents derived from flavonoids.

## Introduction

Each year, more than 600,000 people die from colorectal cancer (CRC) and 1.25 million people are diagnosed with this disease. The surgical removal of cancer by operation is the traditional therapy for all stages of CRC; however, many patients have unresectable tumors and go on to develop metastases [1]. Therefore, novel therapeutic agents for treating CRC are urgently required.

Accumulating evidence has demonstrated that inflammation is a critical component of tumor progression [2]; sites of infection, chronic irritation and inflammation could be high risk areas to develop into cancer. The close connection between Inflammatory bowel diseases (IBDs) and colon cancer has been proposed since 1925 and is still a powerful case to prove the relationship between inflammation and cancer [3, 4]. Previous studies have reported that pro-inflammatory factors of the innate and adaptive immune systems, including IL-6 [5] and TNF-α [6], could contribute to the development and growth of colon neoplasia. NF-κB, which is one of many downstream targets of TNF receptor 1 activation, is likely to play a prominent role in colitis-associated tumorigenesis because aberrant NF-κB activation was detected in > 50% of colorectal and colitis-associated tumors and mouse studies [7]. Taken together, these findings suggest a compelling role for inflammation in colon carcinogenesis.

**Fig 1:**
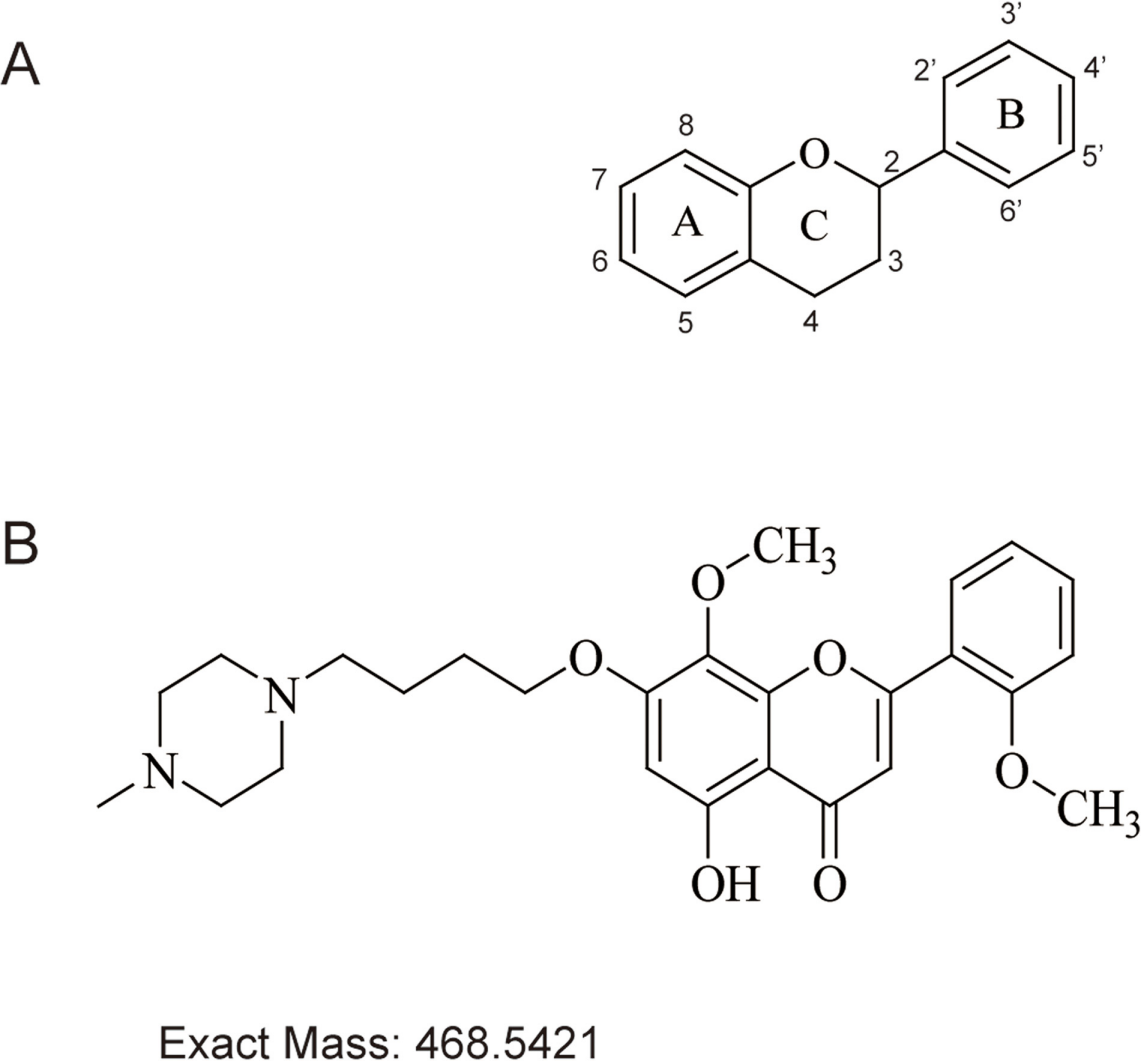
The chemical structures of flavone and LZ-207. (A) Chemical structure of the 15-carbon flavone backbone. (B) Chemical structure of LZ-207 (C_26_H_32_N_2_O_6_, MW = 468.5421).

Natural flavonoids are widespread in the human diet and plants, include all citrus fruits, blueberries, parsley, onions, black tea, green tea, red wine and bananas [8]. These compounds are low molecular weight substances that are based on a common three-ring structure with different substitutions [9]. Since the French paradox left the impression that much of France’s lower incidence of cardiac disease associated with the country’s high levels of red wine consumption, flavonoids from red wine have become a focus of cancer research studies [10]. The potential beneficial properties of flavonoids include antioxidant, antiatherosclerotic, anti-inflammatory, antithrombogenic, antiosteoporotic, and antiviral effects [10]. Recently, the antitumor effects of flavonoids have also been recognized [11]. Many flavonoids, such as quercetin [12], silymarin [13] and luteolin [14], exert antitumor activity against various cancer cell lines, suggesting that these flavonoids are promising agents for cancer prevention and warrant further study. Flavonoids are phenyl-substituted chromones (benzopyran derivatives) that consist of a 15-carbon basic skeleton (C6-C3-C6) (Fig 1A) with a chroman (C6-C3) nucleus (the benzo ring A and the heterocyclic ring C) and with a phenyl group (the aromatic ring B) normally substituted at the 2-position [15]. In recent years, wogonin, which is a flavonoid, has received increasing attention for its antitumor activities in hepatoma [16], breast carcinoma [17], gastric cancer [18], cervical carcinoma [19], and leukemia [20, 21]. Many wogonin derivatives have been synthesized to have better water solubility and druggability, and some of these synthesized derivatives have shown potential antitumor effects. For example, LYG-202, which is a wogonin derivative, induces apoptosis in human hepatocellular carcinoma HepG2 cells via inducing the ROS-mitochondria pathway [22]. LYG-202 also induces cell cycle arrest and apoptosis in human colorectal carcinoma HCT116 cells via its regulation of p53 and p21WAF1/Cip1 [23]. Another wogonin derivative, LW-214, has potent antitumor activity in human breast cancer MCF-7 cells by down-regulating Trx-1 and by activating the JNK pathway, ultimately inducing mitochondria-mediated apoptosis [24]. In this work, we focused on LZ-207, which is a newly synthesized flavonoid with a structure similar to that of wogonin. A methoxy group in LZ-207 is substituted at the 6’-position, and a piperazine substitution occurs at the 7’-position (see Fig 1B). These substitutions improve the water solubility (Table 1) and druggability of LZ-207 compared with other flavonoid family members. Therefore, we were interested in examining the antitumor effects of LZ-207 on colitis-associated cancers and in revealing the interactions between inflammatory cells and tumor cells. In this paper, we studied the growth-inhibitory effects of LZ-207 in HCT116 cells and the inhibitory effects of LZ-207 on inflammatory processes in THP-1 monocytes. We also examined the inhibitory effects of LZ-207 on the inflammatory response elicited by culturing HCT116 cells with LPS-induced human monocyte THP-1 cells.

**Table 1:** The water solubility of wogonin and LZ-207.

	Determine wavelength (nm)	Solubility (μg/ml)
**Wogonin**	277	0.665
**LZ-207**	277	83.828

## Materials and Methods

### Reagents

LZ-207 (purity > 99%), which was obtained from Dr. Zhiyu Li (China Pharmaceutical University, China), was dissolved in DMSO to 0.1 M as a stock solution and stored at -20°C. LZ-207 was freshly diluted with cell culture medium to different final concentrations before each experiment. The final DMSO concentration did not exceed 0.1% and had no effects on cell growth and differentiation.

Lipopolysaccharides(LPSs), 3-(4,5-dimethylthiazol-2-yl)-2,5-diphenyltetrazolium bromide (MTT) and 4’,6-diamidino-2-phenylindole dihydrochloride (DAPI) were purchased from Sigma-Aldrich (St. Louis, MO, USA). An annexin V-FITC apoptosis detection kit, a chemiluminescent electromobility shift assay (EMSA) kit, and human IL-1β and IL-6 enzyme-linked immunosorbent assay (ELISA) kits were purchased from Bender MedSystems Co., Ltd. (Burlingame, CA, USA), Beyotime Institute of Biotechnology (Nanjing, China), and KeyGen Biotech Co., Ltd. (Nanjing, China), respectively.

Primary β-actin antibody was obtained from Boster Biological Technology, Ltd. (Wuhan, China) and used at a 1:20,000 dilution. Primary Akt, Bax, caspase-3, caspase-8, caspase-9, ERK, IκBα, NF-κB, JNK, p38, p-Akt, p-ERK, p-JNK, p-p38 antibodies were obtained from Santa Cruz Biotechnology (Santa Cruz, CA, USA) and were all used at a 1:500 dilution. Primary Bcl-2, IKKα/β, PARP, p-IKKα/β, p-IκBα antibodies were obtained from Cell Signaling Technology (Beverly, MA, USA) and were all used at a 1:1,000 dilution. IRDye 800-conjugated secondary antibodies were obtained from Rockland, Inc. (Philadelphia, PA, USA) and used at a 1:15,000 dilution.

### Cell culture

Human colon carcinoma HCT116 cells, human colorectal cancer SW1116 and HT29 cells, human umbilical vein endothelial HUVEC cells, normal human lung fibroblasts MRC5 cells and human acute monocytic leukemia THP-1 cells were obtained from the cell bank of the Chinese Academy of Sciences (Shanghai, China). HCT116, HT29, SW1116, HUVEC, MRC5 and THP-1 cells were cultured in either McCoy’s 5A medium or DMEM medium (Gibco, Invitrogen Corporation, NY, USA). All the media were supplemented with 10% heat-inactivated fetal bovine serum (FBS) (Gibco, Invitrogen Corporation, NY, USA), 100 U/ml benzyl penicillin, and 100 μg/ml streptomycin at pH 7.4, and cells were maintained in a CO_2_ incubator (Thermo Forma, Thermo Fisher Scientific, Inc., MA, USA) with a humidified atmosphere of 5% CO_2_ at 37°C [25, 26].

### Cell viability assay

The SW1116 cells, HT29 cells, HCT116 cells, HUVEC cells, MRC5 cells and THP-1 cells were plated in 96-well plates with 100 μl of the appropriate medium at an optimal density. The cells were treated with different concentrations of LZ-207 and incubated at 37°C with 5% CO_2_ for 24, 48 and 72 h. Subsequently, 20 μl of MTT solution (5 mg/ml) was added to each well and incubated at 37°C with 5% CO_2_ for another 4 h. Then, the supernatants were discarded, and 100 μl of DMSO was added to each well. The plates were shaken for 2 min to ensure total solubility of the formazan crystals, and cell viability was determined based on the mitochondrial conversion of MTT to formazan. The absorbance (A) was measured at 570 nm using a Universal Microplate Reader (EL800, BioTek Instruments Inc.). The inhibition ratio (%) and survival rate (%) were calculated using the following equations:

$$\text{Inhibitory ratio}\;\left(\%\right)=\frac{{\text{A}}_{\text{Control}}-{\text{A}}_{\text{Treated}}}{{\text{A}}_{\text{Control}}}\times 100\%$$

$$\text{Survival ratio}\;\left(\%\right)=\frac{{\text{A}}_{\text{Treated}}}{{\text{A}}_{\text{Control}}}\times 100\%$$

A_Treated_ and A_Control_ were the average absorbance values of three parallel experiments from the treated and control groups, respectively.

The IC_50_, which is the concentration that caused 50% inhibition of cell viability, was calculated using the logit method [24].

### DAPI staining assay

Cell nuclei were visualized with DAPI staining, which emits blue fluorescence upon binding to AT regions of DNA, to detect any morphological evidence of apoptosis. After LZ-207 treatment for 24 h, HCT116 cells were fixed with cold 4% paraformaldehyde for 30 min, washed twice with phosphate-buffered saline (PBS), and then permeabilized with 0.3% Triton X-100 for 20 min at room temperature. After being washed twice with PBS, the cells were incubated with DAPI (1 μg/ml) for 10 min and then observed using fluorescence microscopy (Olympus, Japan) with a peak excitation wavelength of 340 nm [27].

### Annexin V/PI double staining assay

Apoptosis-mediated tumor cell death was examined using a double staining method with a FITC-labeled Annexin V/PI Apoptosis Detection Kit according to the manufacturer’s instructions. HCT116 cells in McCoy’s 5A medium containing 10% FBS were cultured in 6-well plates at a comfortable density for 24 h. After LZ-207 (5, 10, or 20 μM) treatment for another 24 h, the cells were harvested, washed twice with cold PBS, and then resuspended in 500 μl of binding buffer. Next, 5 μl of Annexin V and 5 μl of PI were successively added to each tube, mixed gently and kept on ice for 10 min in the dark. Data acquisition and analysis were performed using a Becton Dickinson FACS Calibur flow cytometer with FlowJo 7 software with excitation/emission at 488/530 nm. The left lower section of the fluorocytograms (An−, PI−) represents normal cells, the right lower section of the fluorocytograms (An+, PI−) represents early and mid-apoptotic cells, and the right upper section of the fluorocytograms (An+, PI+) represents late apoptotic cells [28, 29].

### Mitochondrial transmembrane potential (ΔΨm) assessment

Mitochondrial transmembrane potential (ΔΨm) changes could be indicated by JC-1, a fluorescent dye from KeyGEN JC-1 Apoptosis Detection Kit (China). After LZ-207 (5, 10, or 20 μM) treatment for 24 h, HCT116 cells were harvested, washed with PBS, resuspended in JC-1 working buffer. After incubating at 37°C with 5% CO_2_ for 30 min, the cells were washed twice with incubation buffer and analyzed by flow cytometry and software FlowJo 7 with settings of FL1 (FITC, green) and FL2 (PI, red) [30].

### Preparation of mitochondrial and cytosolic fractions

The mitochondrial and cytosolic fractions from cells were prepared using KeyGEN mitochondria isolation kit (China) according to the manufacturer’s instructions. After LZ-207 (5, 10, or 20 μM) treatment for 24 h, the HCT116 cells were harvested, washed with PBS, resuspended in cold lysis buffer, and homogenized on ice water with a tight pestle. Then the cells were transferred to medium buffer and centrifuged for 10 min at 4°C (1200 g). The supernatant was collected and centrifuged again for 10 min at 4°C (7000 g) to obtain the mitochondria (pellet) and cytosol (supernatant) fractions. Mitochondria and cytosol fractions of HCT116 cells were stored at -80°C [31].

### Immunofluorescence

HCT116 cells were seeded onto glass coverslips in 6-well plates for 24 h and treated with LZ-207 (5, 10, or 20 μM) with or without LPS (10 μg/ml) for another 24 h. Then, the cells were rinsed three times with PBS for 5 min each, fixed with cold 4% paraformaldehyde for 30 min, rinsed three times with PBS for 5 min each, permeabilized with 0.3% Triton X-100 for 20 min, blocked with 5% BSA for 60 min and incubated with primary NF-κB antibody (diluted 1:200) overnight at 4°C. After rinsing three times with PBS containing 0.01% Tween 80 for 5 min each, the cells were stained with FITC-labeled secondary goat anti-mouse IgG antibody (1:100) at 37°C for 1 h in the dark. Then, the cells were rinsed three times with PBS for 5 min each and stained with DAPI. The images were captured with an Olympus FV1000 confocal microscope [32].

### Preparation of whole cell lysates and cytosolic and nuclear extracts

Briefly, the cells were cultured to approximately 80–90% confluence and treated with LZ-207 at the indicated concentrations with or without LPS (10 μg/ml) for 24 h. The whole cell lysates were prepared as previously described [33]. Nuclear and cytosolic protein extracts were prepared using a Nuclear/Cytosol Fractionation Kit according to the modified manufacturer’s protocol below. After washing twice with PBS, the cells were collected in tubes with PBS by scraping with a cell scraper and centrifuged for 5 min at 4°C (600 g). Then, we removed the supernatant, gently resuspended the cell pellet with cytosolic extraction buffer and incubated this suspension on ice for 15 min, followed by a 15 min centrifugation at 4°C (14,000 g). The supernatant (cytoplasmic fraction) was carefully transferred to a clean, pre-chilled tube and stored at -80°C for later use. Then, the same volume of cytosolic extraction buffer was added to the pellet again, and the same steps were repeated as before, except the supernatant was discarded last. The nuclear pellet was resuspended in nuclear extraction buffer, kept on ice for 30 min, and then centrifuged for 15 min at 4°C (12,000 g). The supernatant (nuclear protein extract) was carefully transferred to a clean, pre-chilled tube and stored at -80°C [33].

### EMSA

HCT116 cell nuclear proteins were extracted as described previously. EMSA was performed using a non-radioactive (biotin labeled) gel shift assay according to the manufacturer’s instructions. Briefly, oligonucleotide probes were synthesized, annealed, and labeled using a biotin 3’-end DNA labeling kit (Pierce). Following the manufacturer’s protocol, prepared DNA-protein complexes were resolved on a 6% non-denaturing polyacrylamide gel in a 0.5× Tris-borate-EDTA buffer at 380 mA for 1 h and then transferred to a nylon membrane. Finally, the gel shift of biotin-labeled DNA was visualized by chemiluminescence using a Bio-Rad infrared system and a chemiluminescent EMSA kit [34].

### Western blot analysis

The whole cell lysates and cytosolic and nuclear extracts from treated cells were prepared as described above, and the protein concentration was measured using a BCA assay with a Varioskan multimode microplate spectrophotometer (Thermo Fisher Scientific, Inc., Waltham, MA, USA) at 562 nm. Equal amounts of protein samples were prepared, separated by SDS-PAGE and transferred onto nitrocellulose (NC) membranes. The membranes were blocked with 1% BSA in PBS at 37°C for 1 h and then incubated with the indicated primary antibodies overnight at 4°C, followed by incubation with IRDye 800-conjugated secondary antibodies for 1 h at 37°C. Detection was performed using the Odyssey Infrared Imaging System (LI-COR Inc., Lincoln, NE, USA). All blots were stripped and reprobed with polyclonal anti-β-actin antibody to ascertain equal loading of proteins [35].

### Cytokine quantification by ELISA

Human IL-6 and IL-1β ELISA kits were used according to the manufacturer’s instructions to quantify the expression of secreted IL-6 and IL-1β cytokines in the supernatants of treated THP-1 cells. The THP-1 cells were treated with LZ-207 (5, 10, or 20 μM) with or without LPS (10 μg/ml). Each experiment was repeated three times. The cytokine levels are expressed in pg/ml. The cytokines were not detected in the fresh medium used to culture these cells [36].

### RNA extraction and quantitative real-time PCR (qPCR) assay

Total RNA from treated THP-1 cells was extracted using a phenol/chloroform method with TRIzol reagent (Invitrogen). Reverse transcription (RT) was performed using equal amounts of mRNA, and quantitative real-time PCR (qPCR) was performed following the Takara kit protocol (Takara, Takara Bio Inc., Otsu, Shiga, Japan). cDNA was also collected for real-time quantitative reverse transcription-PCR (qRT-PCR), which was performed using a Chromo4instrument (Bio-Rad, Berkeley, CA, USA) with SYBR Green Master Mix (Applied Biosystems, CA, USA). The relative amount of target mRNA was determined using the comparative threshold cycle (Ct) method by normalizing target mRNA Ct values to those values for β-actin (△Ct) [37]. The primer sequences used were as follows:

IL-6-sense: 5’-TGTAGTGAGGAACAAGCCAGAG-3’;

IL-6-antisense: 5’-TACATTTGCCGAAGAGCC-3’;

IL-1β-sense: 5’-AGGCTGCTCTGGGATTC-3’;

IL-1β-antisense: 5’-GCCACAACAACTGACGC-3’;

β-actin-sense: 5’-CTGTCCCTGTATGCCTC T-3’;

β-actin-antisense: 5’-ATGTCACGCACGATTTCC-3’

### Co-culture of HCT116 cells with THP-1 cells

HCT116 cells were seeded in 6-well plates at 4 × 10^4^ cells per well and were allowed to grow to approximately 80% confluence. THP-1 cells were collected by centrifugation (600 g for 10 min, washed, and resuspended at a final concentration of 2 × 10^5^ cells/ml) and added to HCT116 cells. Then, the co-culture system was left untreated, activated with LPS, or treated with LZ-207 together with LPS. After 24 h, the co-culture system was stopped, and the cell culture media were removed. The HCT116 cells were washed twice with cold PBS (pH = 7.4), and cell viability was measured using MTT assays as described above [38, 39].

### Antitumor effects on HCT116 nude mice xenografts

The animal experiment was performed on the basis of standard operation procedure established by the State Food and Drug Administration (SFDA) in China. Athymic BALB/c nude mice, female, 35–42 days old with weight ranging from 18 to 22 g were obtained from Shanghai Institute of Materia Medica, Chinese Academy of Sciences. The specific pathogen free nude mice were raised in a controlled environment (23 ± 2°C, 55 ± 5% humidity, 12h light + 12h dark / day) and fed with standard laboratory food and water. 1 × 10^6^ HCT116 cells were injected into the right axilla of each nude mice to establish the animal model. After 12 days, micrometer calipers were used to determine tumor size. Nude mice with similar tumor volume were selected and randomly separated into 5 groups: 0.9% saline control group (12 nude mice), 5-Fu 30 mg/kg positive group (6 nude mice), LZ-207 20 mg/kg group (6 nude mice), LZ-207 10 mg/kg group (6 nude mice) and LZ-207 10 mg/kg group (6 nude mice). The nude mice received intravenous injection every three days. Tumor volume and body weight were measured also every three days. After 21 days treatment, all nude mice were sacrificed, peripheral blood was collected; tumors were removed and weighted; hearts, livers, spleens, lungs and kidneys were separated. Tumor volume (TV) was calculated using the following equation: TV (mm^3^) = D/2 × d^2^, where D and d are the longest and the shortest diameters of tumor, respectively [27].

### Statistical analysis

All data are presented as the mean ± standard deviation (SD) from triplicate experiments performed in a parallel manner, unless otherwise indicated. All data comparisons were made using Student’s *t*-tests and were considered statistically significant at *P < 0.05 and **P < 0.01.

## Results

### LZ-207 inhibits tumor cell viability

**Fig 2:**
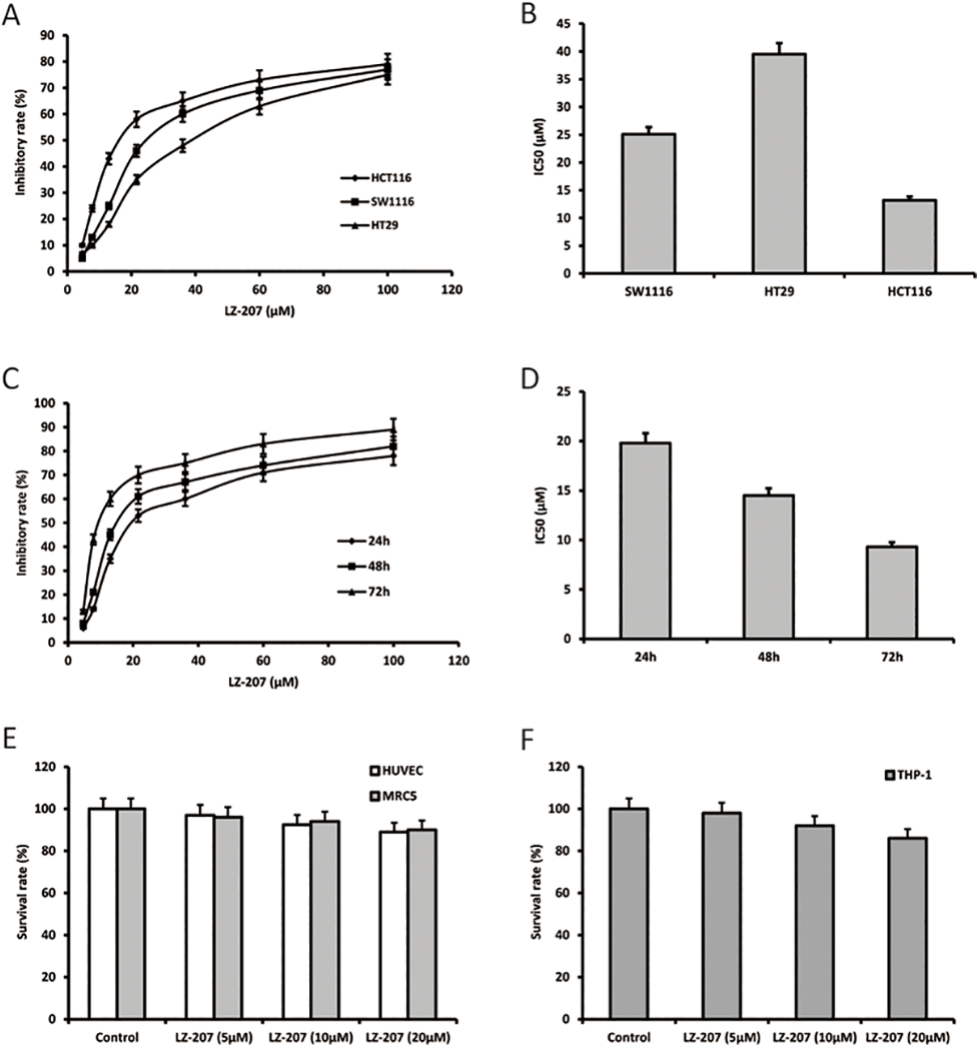
LZ-207 inhibits the viability of various cancer cell lines. (A) The inhibitory effect of a 48 h treatment with LZ-207 on SW1116, HT29 and HCT116 cells. (B) IC_50_ values of a 48 h treatment with LZ-207 in SW1116, HT29 and HCT116 cells. (C) HCT116 cells were treated with different concentrations of LZ-207 for 24, 48 and 72 h. (D) IC_50_ values of 24, 48 and 72 h LZ-207 treatments in HCT116 cells. (E) The survival rate of a 48 h treatment with LZ-207 on HUVEC and MRC5 cells. (F) The survival rate of a 48 h treatment with LZ-207 on THP-1 cells. The data are presented as the mean ± SD (n = 3).

MTT assays were used to examine the effect of various concentrations of LZ-207 on cell viability of tumor cell lines (SW1116, HT29 and HCT116 cells), benign cell lines (HUVEC and MRC5 cells) and THP-1 cells. After a 48 h treatment, LZ-207 effectively inhibited the viability of SW1116, HT29, and HCT116 cells (Fig 2A), with IC_50_ values of 25.1 ± 0.86, 39.5 ± 1.13, and 13.2 ± 1.49 μM, respectively (Fig 2B). As shown in Fig 2C, HCT116 cells exhibited time- and dose-dependent sensitivity to LZ-207. The IC_50_ values for 24, 48 and 72 h LZ-207 treatments in HCT116 cells were 19.81 ± 0.75, 14.58 ± 0.42, and 9.32 ± 0.50 μM, respectively (Fig 2D). Therefore, the HCT116 cell line was chosen for all subsequent experiments using 5, 10 and 20 μM LZ-207 treatments for 24 h. Three dosages of LZ-207 also showed no obvious effect on the survival rate of benign cells and THP-1 cells which will be used in the co-culture system below.

### LZ-207 induces apoptosis in HCT116 cells

**Fig 3:**
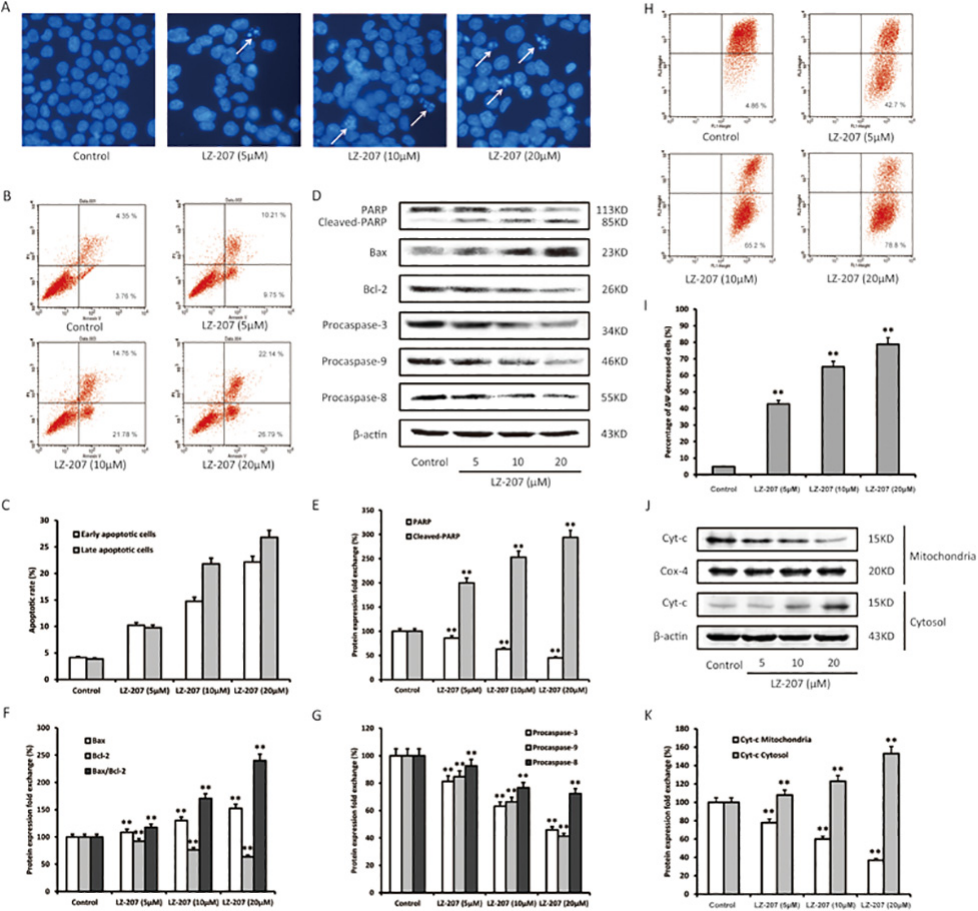
LZ-207 induces apoptosis in HCT116 cells. HCT116 cells were treated with 5, 10 or 20 μM LZ-207 for 24 h. (A) Morphologic changes of the nucleolus were observed using fluorescence microscopy (400×). Cells were examined for the presence of apoptotic bodies and nuclear pyknosis. (B) Annexin V/PI double staining assay of HCT116 cells. The *Y*-axis shows the PI-labeled population, and the *X*-axis shows the FITC-labeled Annexin V-positive cells. (C) The apoptotic rates of HCT116 cells induced by LZ-207. (D) Western blotting analysis of PARP, Bax, Bcl-2, procaspase-3, procaspase-9, and procaspase-8 in HCT116 cells treated with LZ-207. (E-G) Densitometric analysis represents the relative fold change in protein expression. (H-I) The change of ΔΨm was detected by using JC-1 staining and analyzed by flow cytometry in LZ-207 treated HCT116 cells. (J-K) Western blot analysis of Cytochrome c in mitochondria and cytosol in LZ-207 treated HCT116 cells. The data are presented as the mean ± SD (n = 3). *P < 0.05, **P < 0.01, significant difference compared with the control.

The morphology of HCT116 cells was severely altered after 24 h of treatment with LZ-207. To examine whether LZ-207 induced apoptosis in HCT116 cells, DAPI staining was used to detect nuclear changes. Fluorescence microscopy showed that control cells were uniformly stained with blue fluorescence, demonstrating the typical homogeneous distribution of chromatin in the nucleolus. By contrast, HCT116 cells treated with LZ-207 emitted a brighter fluorescence, which is indicative of early apoptosis because of chromatin condensation and nucleolus pyknosis (Fig 3A).

LZ-207-induced apoptosis in HCT116 cells was further confirmed using Annexin V/PI staining assays. Following treatment with 5, 10, or 20 μM LZ-207 for 24 h, the early apoptotic rates in control and treated HCT116 cells were 4.35, 10.21, 14.76, and 22.14%, respectively, and the late apoptotic rates were 3.76, 9.75, 21.78, and 26.79%, respectively (Fig 3B and 3C). These results demonstrated that LZ-207 treatment induces both early and late apoptosis in a concentration-dependent manner.

Western blots showed that the protein level of cleaved PARP increased with increasing concentrations of LZ-207, whereas the protein level of intact PARP decreased, confirming the ability of LZ-207 to induce apoptosis as part of its antitumor effect on HCT116 cells (Fig 3D and 3E). We also found that Bcl-2 expression decreased after HCT116 cells were treated with LZ-207 for 24 h, whereas Bax expression increased, leading to a significant increase in the Bax/Bcl-2 ratio (Fig 3F). We further detected that procaspase-3 and procaspase-9 expression significantly decreased after LZ-207 treatment, indicating that this mitochondrial pathway may be involved in LZ-207-induced apoptosis (Fig 3G). During the mitochondria-mediated apoptosis, depolarization of the mitochondrial transmembrane potential (ΔΨm) accompanied by releasing cytochrome c from mitochondrial into the cytosol, which triggers a caspase-dependent apoptosis. There was an obvious increase in green fluorescence of JC-1 monomers in LZ-207 treated HCT116 cells, indicating depolarization of the mitochondrial transmembrane potential (ΔΨm) (Fig 3H and 3I). We also found that the expression of Cyt-c decreased in mitochondria while increased in cytosol in LZ-207 treated HCT116 cells (Fig 3J and 3K). Meanwhile, the slight degradation of procaspase-8 suggested that an extrinsic apoptosis pathway might also correlate with LZ-207-induced apoptosis (Fig 3G).

### Inhibitory effect of LZ-207 on LPS-induced NF-κB p65 activation in HCT116 cells

**Fig 4:**
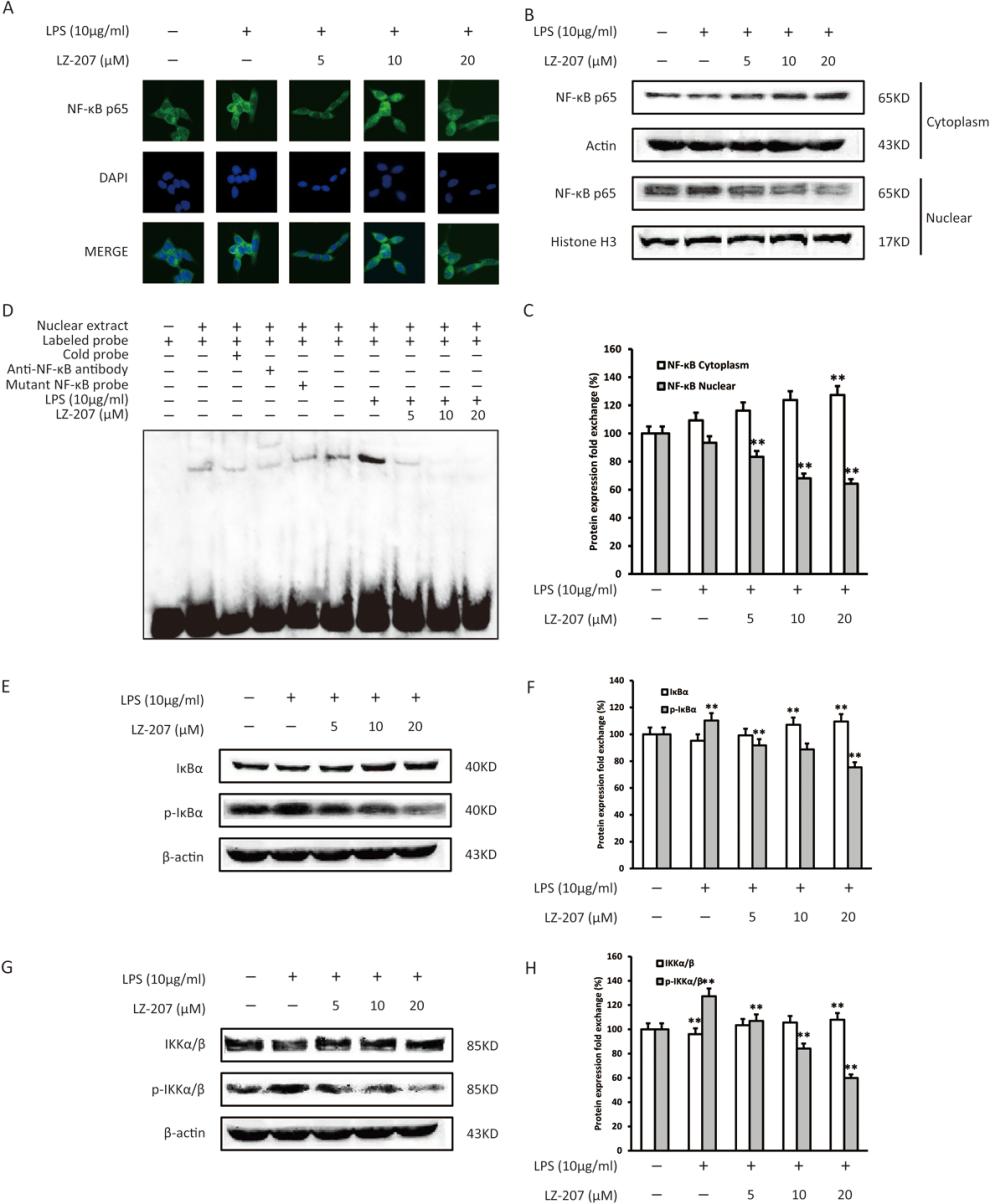
Effect of LZ-207 on NF-κB expression in LPS-treated HCT116 cells. HCT116 cells were treated with or without LZ-207 in the presence of LPS for 1 h. After isolating the nuclear and cytoplasmic extracts, (A) NF-κB p65 levels were determined by immunofluorescence, and (B-C) NF-κB p65 translocation was measured via Western blotting. (D) LZ-207 suppressed LPS-induced NF-κB DNA binding activity in a concentration-dependent manner, as detected via EMSA assay. (E-H) Western blotting analysis of the phosphorylation of IκB and IKK in HCT116 cells treated with or without LZ-207 in the presence of LPS for 1 h. The data are presented as the mean ± SD (n = 3). *P < 0.05, **P < 0.01, significant difference compared with the control.

The NF-κB family of transcription factors regulates the expression of multiple genes, including inflammation-associated genes. Thus, the dysregulation of NF-κB signaling is a marker of cancer development. Using immunofluorescence confocal microscopy (Fig 4A), we observed that LZ-207 treatment inhibited NF-κB p65 nuclear translocation in HCT116 cells. We quantified the amount of NF-κB p65 in the nuclear fraction of LZ-207-treated HCT116 cells via Western blot analysis, the expression of nuclear NF-κB increased with 10 μg/ml LPS treatment in HCT116 cells, as shown in Fig 4B and 4C; however, this LPS-induced NF-κB nuclear translocation was suppressed by LZ-207 treatment. Using EMSA assays, we also demonstrated that LZ-207 suppressed LPS-induced NF-κB DNA binding activity in a dose-dependent manner in HCT116 cells (Fig 4D).

Because NF-κB activation results from the rapid phosphorylation, ubiquitination, and ultimately proteolytic degradation of IκB, we examined the effect of LZ-207 on the expression of phosphorylated IκBα in LPS-induced HCT116 cells via Western blot. LZ-207 significantly inhibited IκBα phosphorylation compared with the control but had no effect on IκBα protein expression (Fig 4E and 4F). Because IκB degradation is primarily dependent on IKK activation, next, we examined the effect of LZ-207 on IKK activation and found that LZ-207 significantly suppressed LPS-induced IKKα/β phosphorylation in HCT116 cells (Fig 4G and 4H).

### LZ-207 inhibits signaling pathways upstream of NF-κB in HCT116 cells

**Fig 5:**
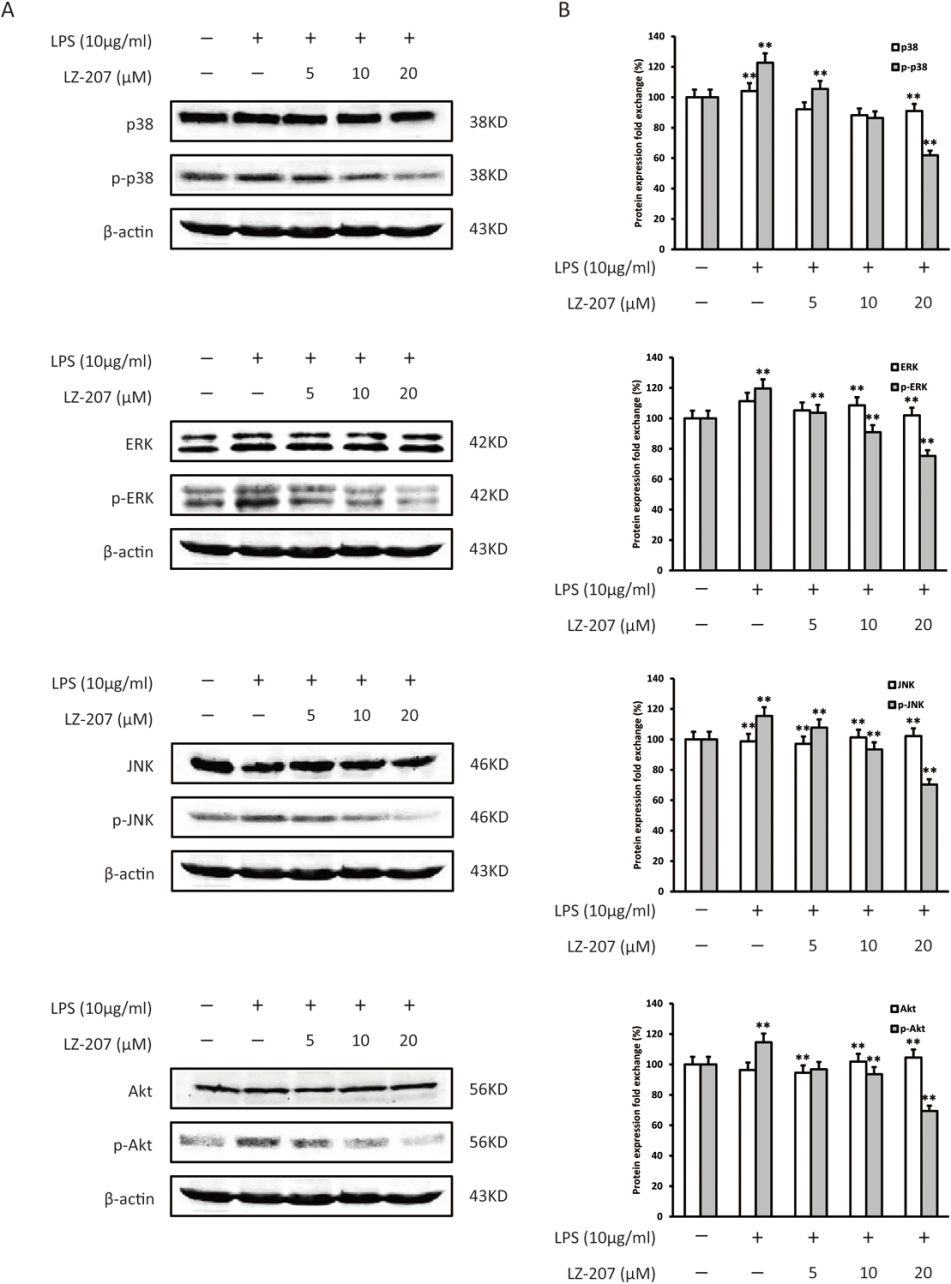
Effect of LZ-207 on the signaling pathway upstream of NF-κB in LPS-treated HCT116 cells. HCT116 cells were treated with or without LZ-207 in the presence of LPS for 1 h. (A) Western blotting analysis of p38, p-p38, ERK, p-ERK, JNK, p-JNK, Akt, p-Akt protein expression. (B) Densitometric analysis represents the relative fold change in protein expression. The data are presented as the mean ± SD (n = 3). *P < 0.05, **P < 0.01, significant difference compared with the control.

Cells sense changes in their environment via the activation of signal transduction pathways that direct biochemical programs to mediate proliferation and survival. The mitogen-activated protein kinase (MAPK) family and Akt signaling pathways can regulate these fundamental cellular processes through the induction of IKK-dependent NF-κB activation. We examined the expression of factors upstream of NF-κB activation, including p38 MAPK, ERK1/2, JNK and Akt, following LZ-207 treatment in LPS-induced HCT116 cells. As shown in Fig 5A and 5B, LZ-207 inhibited the LPS-induced phosphorylation of p38 MAPK, ERK1/2, JNK and Akt, whereas the expression levels of p38 MAPK, ERK1/2, JNK, and Akt were not changed.

### LZ-207 suppresses pro-inflammatory cytokines in LPS-induced THP-1 cells

**Fig 6:**
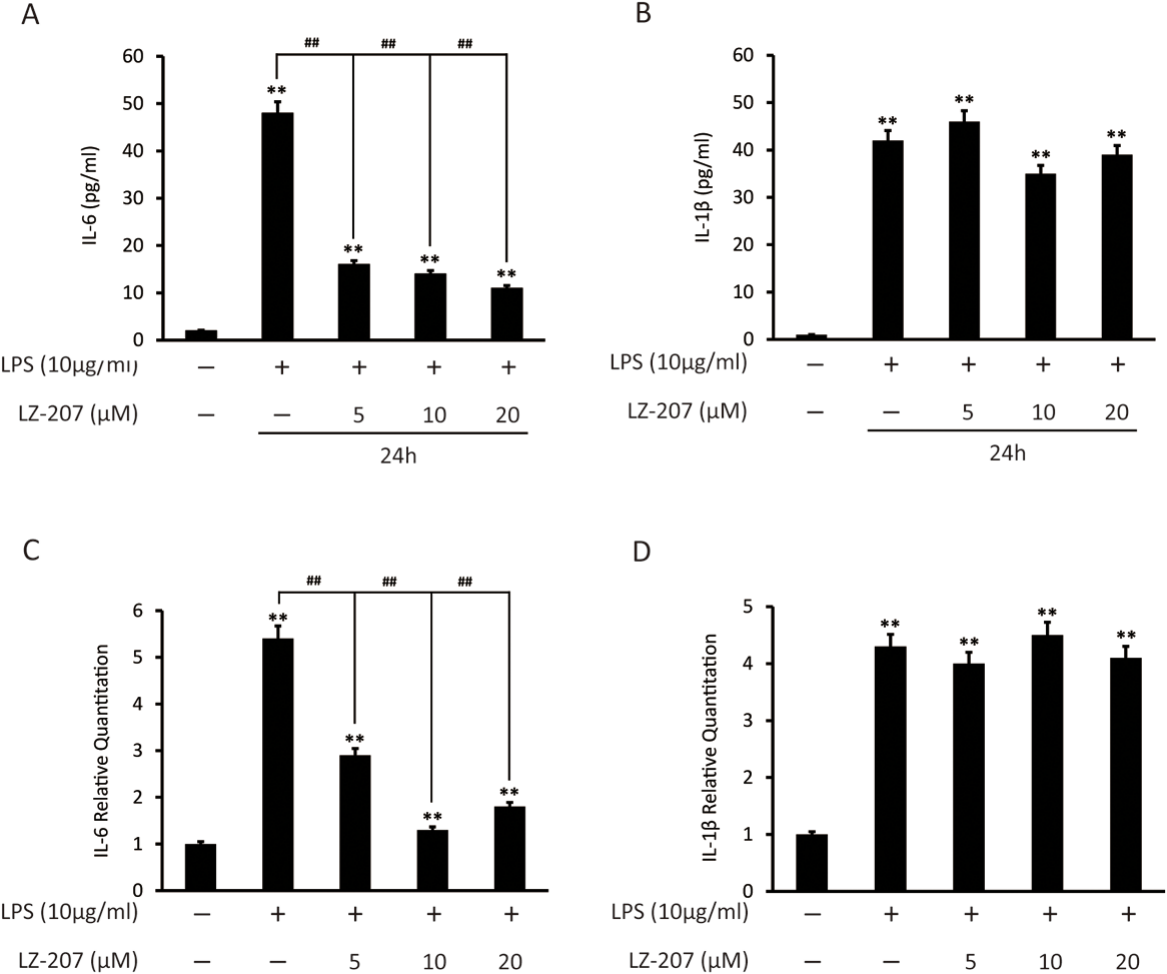
Effect of LZ-207 on the expression of inflammation-related genes in THP-1 human monocyte cells. (A-B) The levels of IL-6 and IL-1β in the culture medium were measured using ELISA kits following treatment with LPS in the presence or absence of different concentrations of LZ-207 in THP-1 cells. (C-D) Total RNA was obtained from THP-1 cells stimulated with LPS in the presence or absence of LZ-207 using a TRIzol reagent kit. The mRNA levels of IL-6 and IL-1β were measured using RT-PCR. β-actin was used as an internal control. The data are presented as the mean ± SD (n = 3). *P < 0.05, **P < 0.01, significant difference compared with the control.

We examined the paracrine secretion of IL-6 and IL-1β in the culture media of LPS-induced THP-1 cells following LZ-207 treatment using ELISA assays. As shown in Fig 6A and 6B, LZ-207 significantly inhibited the LPS-induced increased expression of IL-6 but had no effect on the expression of IL-1β.

The inhibitory effect of LZ-207 on the expression of pro-inflammatory cytokines in LPS-induced THP-1 cells was also confirmed at the transcriptional level using qPCR assays. As shown in Fig 6C and 6D, the expression of IL-6 mRNA was significantly suppressed by LZ-207 in LPS-induced THP-1 cells, whereasLZ-207 showed no effect on the expression of IL-1β mRNA.

### Inhibitory effect of LZ-207 on LPS-induced NF-κB p65 activation in THP-1 cells

**Fig 7:**
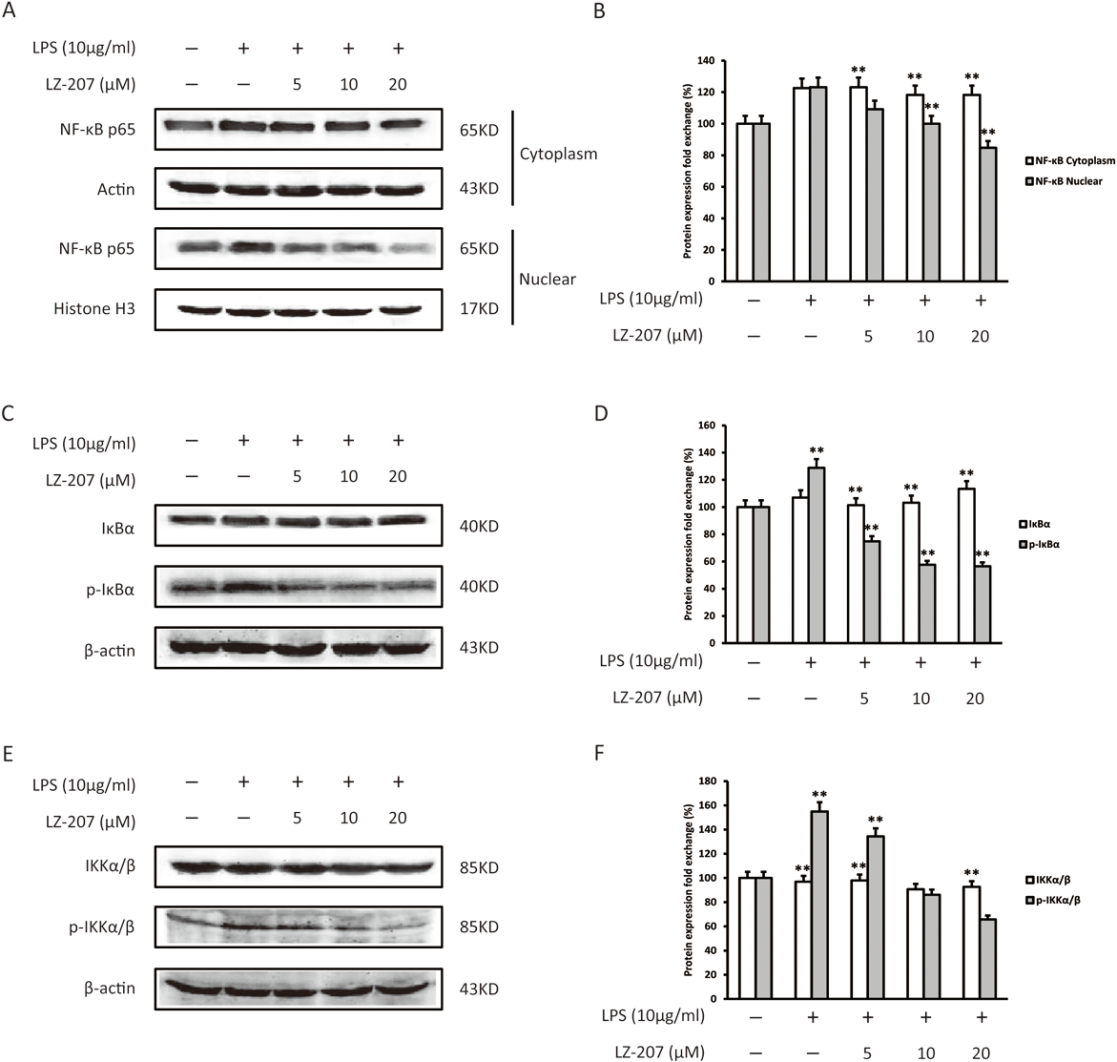
Effect of LZ-207 on the nuclear translocation of NF-κB p65 and on the phosphorylation of IκB and IKK in LPS-treated THP-1 cells. (A-B) The nuclear and cytoplasmic extracts were separated and analyzed for NF-κB expression via Western blot. (C-F) Western blotting analysis of the phosphorylation of IκB and IKK in THP-1 cells treated with or without LZ-207 in the presence of LPS. The data are presented as the mean ± SD (n = 3). *P < 0.05, **P < 0.01, significant difference compared with the control.

We examined the effect of LZ-207 on NF-κB activation in LPS-induced THP-1 cells via Western blot. As shown in Fig 7A and 7B, LZ-207 significantly inhibited the LPS-induced NF-κB nuclear translocation in THP-1 cells. We also found that incubating THP-1 cells with LPS induced IκBα phosphorylation but that this induction was significantly inhibited by LZ-207 (Fig 7C and 7D). In addition, IKKα/β phosphorylation was also inhibited by LZ-207 in LPS-induced THP-1 cells (Fig 7E and 7F).

### LZ-207 inhibits the proliferation of HCT116 cells co-cultured with THP-1 cells

**Fig 8:**
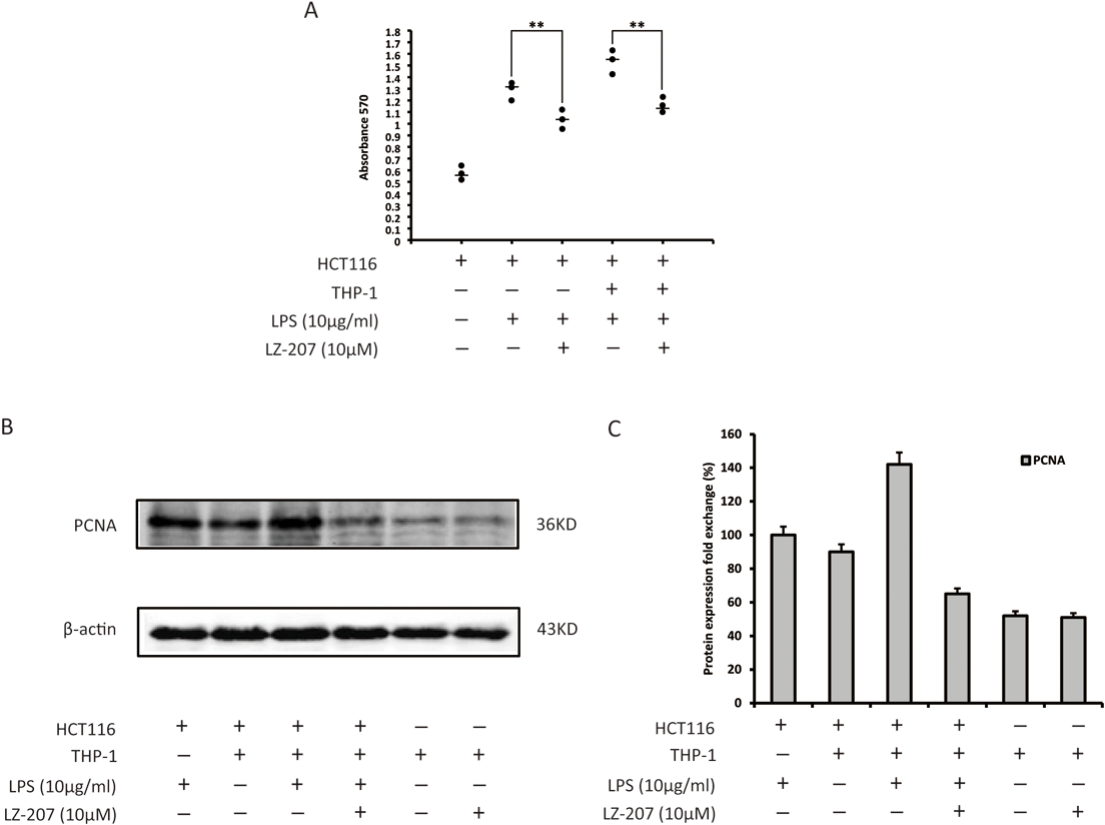
Effect of LZ-207 on HCT116 cell growth when co-cultured with LPS-induced THP-1 cells. (A) HCT116 cells were seeded at a density of 4 × 10^4^ cells/well with or without THP-1 cells stimulated with LPS in the presence or absence of LZ-207. After 24 h, the co-culture was stopped, and the viability of the HCT116 cells was measured via MTT assay. (B-C) Western blotting analysis of PCNA protein levels in co-cultured HCT116 cells. The data are presented as the mean ± SD (n = 3). *P < 0.05, **P < 0.01, significant difference compared with the control.

To study the association between inflammation and tumor promotion in colon cancer, we established a co-culture system using HCT116 cells and THP-1 cells to provide an approximation of physiological conditions. As shown in Fig 8A, LZ-207 significantly inhibited the THP-1-induced proliferation of HCT116 cells. The expression of proliferating cell nuclear antigen (PCNA) was increased in HCT116 cells co-cultured with THP-1 cells; however, this increase was inhibited by LZ-207 treatment (Fig 8B and 8C). Meanwhile, LZ-207 showed no effect on PCNA expression in THP-1 cells (Fig 8B and 8C). These findings suggest that LZ-207 directly suppresses the inflammation-mediated proliferation of HCT116 cells interacting with THP-1 cells in co-culture microenvironments.

### LZ-207 exhibits antitumor effect and low toxicity in vivo

**Fig 9:**
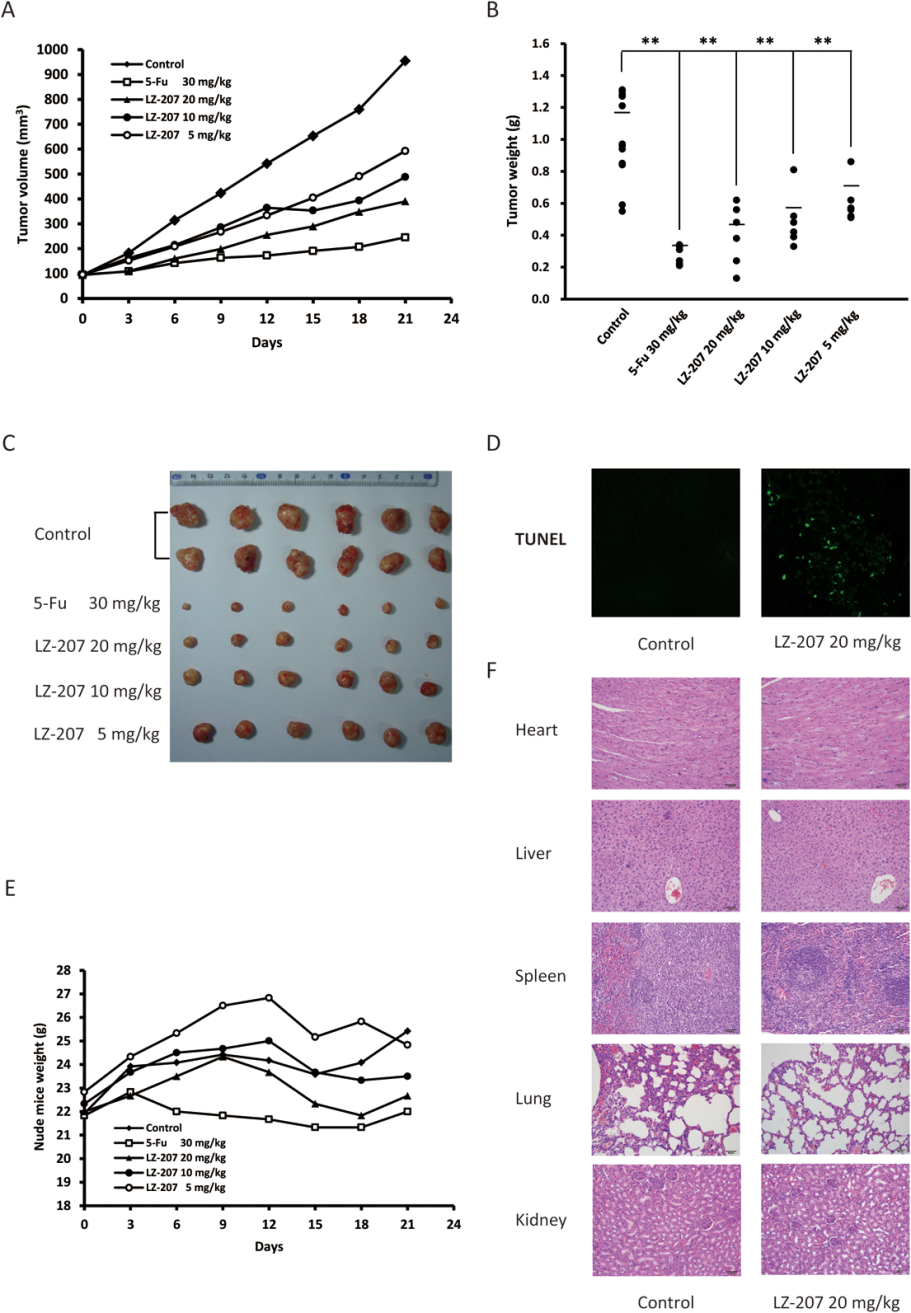
LZ-207 exhibites antitumor effect and low toxicity in vivo. (A) In the nude mice model bearing inoculated HCT116 cells, tumor volume of control, 5-Fu (30 mg/kg) and LZ-207 (20, 10, 5 mg/kg) groups were measured every three days. (B) Tumors from control, 5-Fu (30 mg/kg) and LZ-207 (20, 10, 5 mg/kg) groups were separated and weighted after 21 days treatment. (C) Images of separated tumors from control, 5-Fu (30 mg/kg) and LZ-207 (20, 10, 5 mg/kg) groups. (D) Immunofluorescent TUNEL staining of tumors from control and LZ-207 20 mg/kg groups. (E) Nude mice weight from control, 5-Fu (30 mg/kg) and LZ-207 (20, 10, 5 mg/kg) groups were measured every three days.(F) H&E staining of main organs from control and LZ-207 20 mg/kg groups. The data are presented as the mean ± SD (n = 3). *P < 0.05, **P < 0.01, significant difference compared with the control.

To further evaluate the antitumor effect of LZ-207 in vivo, the nude mice xenografts tumors model bearing inoculated HCT116 cells was established. After 21 days treatment, the tumor volume of control group was 954 ± 272 mm^3^, while the tumor volume of 5-Fu group (30 mg/kg) and LZ-207 groups (20, 10, and 5 mg/kg) were 245 ± 63 mm^3^, 390 ± 134 mm^3^, 488 ± 132 mm^3^, and 592 ± 86 mm^3^, respectively (Fig 9A). As shown in Fig 9B, the tumor weight of control group was 1.01 ± 0.26 g, while the tumor weight of 5-Fu group (30 mg/kg) and LZ-207 groups (20, 10, and 5 mg/kg) were 0.28 ± 0.05 g, 0.41 ± 0.17 g, 0.49 ± 0.15 g, and 0.61 ± 0.12 g, respectively. Both tumor volume and tumor weight measurement exhibited the antitumor effect of LZ-207 in vivo, which was also visually shown in Fig 9C. TUNEL assay was used to examine the apoptotic cells in tumor tissues. As shown in Fig 9D, LZ-207 20 mg/kg group showed enhanced staining intensities compared with control group, indicating LZ-207 treated tumors were going through programmed cell death by apoptosis. Taken together, LZ-207 exhibited antitumor effect through inducing apoptosis in vivo.

To assess the toxicity of LZ-207 in vivo, the nude mice were weighted every three days during the experimental period. There was no obvious variation in the body weight of LZ-207 treated nude mice groups compared with control group (Fig 9E). H&E staining of hearts, livers, spleens, lungs and kidneys also showed no significant morphological change between LZ-207 treated groups and control group (Fig 9F). Meanwhile, hematological parameters obtained from blood routine examination of nude mice were almost in the standard range which showed low toxicity of LZ-207 in vivo (Table 2).

**Table 2:** Hematological parameters of nude mice injected with HCT116 cells.

Hematological parameters	Control	5 mg/kg	10 mg/kg	20 mg/kg	Standard
**White blood cells (10** ^**3**^ **/μl)**	5.54/6.51	4.43/5.73	3.95/4.37	4.82/5.23	3.5–9.5
**Red blood cells (10** ^**3**^ **/μl)**	7.63/7.53	7.91/7.71	7.83/7.58	7.64/7.92	7.51–16.1
**Hemoglobin (g/L)**	136/129	136/141	141/139	146/130	128–161
**Platelet (10** ^**3**^ **/μl)**	330/263	280/300	175/255	312/227	125–350
**Neutrophils (%)**	53.4/49.0	40/47.7	41.3/31.2	33.0/56.2	40–75
**Lymphocytes (%)**	40.4/56.8	47.9/35.2	52.7/52.8	44.1/25.3	20–50
**Monocytes (%)**	5.24/4.04	1.94/4.54	5.54/5.34	2.65/3.48	3–10
**Eosinophils (%)**	0.24/0.04	0.24/0.14	0.04/0.24	0.03/0.26	0.4–8.0
**Basophils (%)**	0.44/0.24	0.14/0.24	0.34/0.24	0.24/0.31	0–1

## Discussion

In this study, we found that the newly synthesized wogonin derivative LZ-207 showed potential antitumor activity in HCT116 human colon cancer cells by inducing mitochondrial-mediated apoptosis and by suppressing pro-inflammatory cytokine secretion from THP-1 human acute monocytic leukemia cells in vitro and in vivo. Specifically, the apoptotic rate reached a maximum value of 48% after LZ-207 (20 μM) treatment. Furthermore, we observed an increase in the Bax/Bcl-2 ratio, activation of caspase-9 and caspase-3, cleavage of PARP, collapse of mitochondrial transmembrane potential (ΔΨm) and release of Cyt-c from mitochondria into cytosol in HCT116 cells suggesting that the intrinsic mitochondrial pathway was involved in mediating LZ-207-induced apoptosis. Together, these results indicated that LZ-207 has a remarkable antitumor activity in HCT116 colon cancer cells.

Increasing evidence suggests that inflammation may contribute to all stages of tumorigenesis [40]. In the stage of tumor initiation, inflammatory microenvironment has been found to promote mutation rates through inducing DNA damage and genomic instability. In the stage of tumor promotion, tumor promoting cytokines secreted by inflammatory cells can stimulate cell proliferation and reduce cell death is considered to be a major mechanism in inflammation-driven tumor promotion. In the stage of tumor metastasis, inflammation is also involved in this critical step through the production of mediators which can promote angiogenesis and cancer cell migration [41, 42]. From this perspective, the inflammatory microenvironment becomes an integral part of the cancer and a potential target for cancer therapy. NF-κB is a key endogenous factor involved in inflammation-induced tumor promotion and progression [43] and has been reported in various human disease and animal models [44]. In the present study, we found that LZ-207 decreased the expression of nuclear NF-κB in a dose-dependent manner in HCT116 cells via inhibiting the phosphorylation of IκBα and IKKα/β. Because the mitogen-activated protein kinase (MAPK) family and Akt signaling pathways can regulate fundamental cellular processes through the induction of IKK-dependent NF-κB activation, we examined the expression of these important upstream factors and found that LZ-207 inhibited LPS-induced phosphorylation of p38 MAPK, ERK1/2, JNK and Akt but that the expression levels of p38 MAPK, ERK1/2, JNK, and Akt were not changed. Therefore, we propose that LZ-207 inhibits cell growth at least partly through suppressing the NF-κB signaling pathway.

Dann et al reported that inhibiting NF-κB activation leads to the repression of the recruitment and activation of immune cells and pro-inflammatory cytokines, such as IL-6 and IL-1β; this repression helps to maintain chronic inflammation and ensure the continuous production of cytokines and growth factors required for the survival and growth of cancer cells [45]. In this study, we found that LZ-207 significantly decreased the expression of IL-6, but not IL-1β, in LPS-induced THP-1 cell culture media. Notably, LPS treatment increased IL-1β secretion; however, this up-regulation was not inhibited by LZ-207 treatment. Further studies are required to elucidate the mechanism of this selective interaction to better understand the relation between inflammation and cancer. Moreover, we found that LZ-207 could suppress the proliferation of HCT116 cells co-cultured with LPS-stimulated THP-1 cells under approximate physiological conditions. Therefore, the inhibition of HCT116 cell growth elicited by LPS-induced THP-1 cells may result from the suppression of IL-6 stimulated proliferation.

In addition, the antitumor effect of LZ-207 in vivo was evaluated in a nude mice model bearing inoculated HCT116 tumor. The results showed that LZ-207 inhibited tumor growth significantly with tumor inhibition rates of 60.07%, 51.12%, 39.69% at the dose of LZ-207 20, 10, 5 mg/kg, respectively. Meanwhile, there was no significant toxicity of LZ-207 in vivo since no obvious changes in the body weight, main organs and hematological parameters were observed between LZ-207 treated groups and control group nude mice. Our study also showed LZ-207 treated groups exerted antitumor effect through inducing apoptosis by TUNEL assay.

In summary, we demonstrated that LZ-207, which is a newly synthesized flavonoid, exhibited an antitumor effect against inflammation-related colon cancer in vitro and in vivo. An understanding of the underlying mechanisms of LZ-207 antitumor activity holds promise for the further development of potential antitumor agents for treating inflammation-related colon cancer.
